# Reply to: Reassessing gas generation from oil thermal cracking associated with the Emeishan Large Igneous Province

**DOI:** 10.1038/s41467-025-59594-5

**Published:** 2025-05-16

**Authors:** Chengsheng Chen, Shengfei Qin, Yunpeng Wang, Greg Holland, Peter Wynn, Wanxu Zhong, Zheng Zhou

**Affiliations:** 1 State Key Laboratory of Deep Earth Processes and Resources, Guangzhou Institute of geochemistry, Chinese Academy of Sciences, 510640 Guangzhou, China; 2 Lancaster Environmental Centre, Lancaster University, Lancaster, LA1 4YQ UK; 3 Research Institute of Petroleum Exploration & Development, PetroChina, 100083 Beijing, China; 4 Department of Earth and Environmental Sciences, The University of Manchester, Manchester, M13 9PL UK

**Keywords:** Solid Earth sciences, Environmental sciences

**replying to** H. Huang *et al*. *Nature Communications* 10.1038/s41467-025-59593-6 (2025)

In their Matters Arising article, Huang et al. raised several concerns that call into question our conclusions from the results reported in our original manuscript (Chen et al.^1^). We clearly acknowledge the importance of discussion on our findings, but at the same time have reservations about their misinterpretations of our data interpretation and modeling processes. We have responded to their four major concerns, including (1) whether the ELIP event made a significant impact on the central area of the Sichuan Basin; (2) whether the ELIP event dominated the destruction of paleo-oil reservoirs in the Sichuan Basin; (3) whether methane gas generation and emissions were overestimated; and (4) uncertainty analysis in the palaeotemperature modeling.

## The ELIP event made a significant impact on the central area of the Sichuan Basin

Huang et al. underestimated our new evidence and related discussion. Their understanding of the impact of the ELIP on the central Sichuan Basin is based on invalid views from petroleum geology alone. An event associated with mantle plume is not simply a heat conduction event, but a complex geodynamic evolution process. Even though the central Sichuan Basin is located in the outer zone of the ELIP, its magmatism and high paleo-heat flow anomaly could still be introduced by the processes like lithosphere extension, plume/asthenosphere upwelling, and decompression melting^2^. Due to topography and later denudation, the absence of basalts does not necessarily mean that the area has not been affected by a volcanic event. In addition, a distance of 100 km is perfectly attainable on a geological scale. Our interpretation of the influence of ELIP on the Sichuan Basin is further supported by a recent publication on the anatomy of the Emeishan mantle plume head by Li et al.^3^, suggesting that narrow finger-like protrusions and plumelets developed outward from the main plume body to the edges of the flatted plume head^3^. These dragged fingers might have fragmented and dispersed into multiple plumelets, which were trapped beneath the thinnest lithosphere relief, and eventually impacted on the outer zone of the ELIP, including the Sichuan Basin^1,3^.

We have no objection to the possibility of multiple hydrothermal activities occurred inside the Sichuan basin strata over geological history, which is the fundamental cause for the different ages from dating results. However, current research suggests that the ELIP event is the most intense tectonic-thermal anomaly of the basin since ultra-high paleo-heat flows (~114 mW/m^2^) during the Late Permian have been recognized for decades^2,4^. Therefore, strong thermal anomalies combined with active tectonics were sufficient to lead to rapid and extensive pyrolysis of organic matter and followed by the release of natural gases. These processes have been considered in recent years, although, their importance has been underestimated in previous studies^1,4,5^.

In our study, the novel combination of noble gas and methane clumped isotope techniques provide strong evidence for settling the debate on the generation and emission of high temperature methane in the Late Permian Sichuan Basin. Solid bitumen associated with rapid cracking of organic matter is clearly the by-product of this process. Obviously, the Emeishan mantle plume, as well as tectonics associated with it, was undoubtedly the only way to connect the mantle and even the deep mantle to the crust in this region since Phanerozoic. In addition to our evidence of high-temperature methane, the latest study suggests a maximum natural gas formation temperature of ~296 °C based on both Δ^13^CH_3_D and ^12^CH_2_D_2_ analysis at the same region^6^. Furthermore, mantle-derived noble gas signatures, i.e. high ^3^He/^4^He ratios, have been detected in the central Sichuan Basin, even considering the high accumulation of ^4^He in the Sichuan craton basin. Although other hydrothermal activities may contribute some primordial ^3^He, they were less likely to provide comparable mantle-derived ^3^He in the same quantity as the ELIP event. In the comments by Huang et al, their arguments support our interpretations and models, unless they can deny the ELIP event itself.

## The ELIP event dominated the destruction of paleo-oil reservoirs in the Sichuan Basin

In their comments, Huang et al. agreed that natural gas generated by burial-dominated heating process cannot fully explain the fact that all exploration and production of natural gas are in the Sinian–Paleozoic reservoirs. Our model of gas generation by abnormal heating event induced by mantle activities is strongly supported by isotopic evidence. At present, the Sinian–Paleozoic petroleum reservoirs in the Sichuan Basin are all dominated by natural gas. This is completely different from petroleum reservoirs with similar ages in the basins experienced deep burial history (e.g., the Tarim Basin, northwest China). Therefore, deep burial alone would not have been able to explain the generally high or over-high maturity and the complete natural gas production in the Sinian–Paleozoic reservoirs in the Sichuan Basin.

Furthermore, large amounts of solid bitumen have been found in the Sichuan basin. This implies a rapid heating process induced by the magma-hydrothermal interaction with the organic material. It is normal for a cratonic basin to contain unevenly distributed solid bitumen, because long-term geological evolution would have allowed it to undergo more complex diagenesis, generation, and alteration processes. Conversely, slow geological burial would lead to low heating rates, which are generally not conducive to the formation of enriched solid bitumen on a large scale. Such formation predominantly corresponds to rapid cracking of enriched oil reservoirs. The pyrolytic products (e.g., oils and condensates) would not remain in place to be completely cracked during the gradual burial at low heating rates. They would have enough time and space to migrate upwards and form various types of oil and gas at the right reservoir conditions.

We have to reiterate that our study provides new evidence for a fundamental and important geological process. Huge volume of high-temperature methane induced by massive volcanic activities has played a key role in global warming. In addition, our findings challenge the traditional understanding of the evolution of source rocks and hydrocarbons in the Sichuan Basin. We suggest that the process of thermal maturation of the Sichuan Basin mainly occurred in the late Permian or slightly later. It related to the ELIP event rather than the burial process later. It is true that the farther away from the mantle plume the smaller the impact it would generate. Re-examining the role of the thermal anomaly on the petroleum-bearing system during the Late Permian in the Sichuan Basin is absolutely necessary.

Regarding rhenium–osmium (Re–Os) dating, we understand that the Re–Os ages of pyrobitumen only represent the last burial alteration that reset the ‘clock’ of pyrobitumen, rather than its primary formation during the ELIP event. This is because pyrobitumen, composed of complex organic mixtures, lacks a fixed composition and does not have a relatively high and stable closure temperature like minerals^7^, leading to its ambiguous closure temperature, uncertain closure conditions, and multi-interpretation^8^. Therefore, prolonged and complex geological processes and reservoir–reburial alterations may significantly affect the construction of Re–Os isochron ages and even reset the timer. Factors here such as significant differences in crude oil components, oil expulsion–migration differentiation, and the input of ELIP-derived mantle materials contribute to this complexity. We believe that these ages may help understand the overall geological processes but cannot be used for denying the influence of ELIP. In addition, Zhu et al.^5^ concluded that the anisotropic pyrobitumen in the reservoirs experienced the highest paleo-temperature over 300 °C during the ELIP event, which coincides with the temperature analysis of the fluid inclusions^5^. Yang et al.^9^ reported a well-preserved mineralogical, fluid inclusion, and geochemical record from an Ediacaran carbonate reservoir (i.e. the Sinian Dengying Formation) in the central Sichuan Basin, and concluded that the pyrolysis of the paleo-oil pools occurred around 258.4 ± 7.3 Ma, which coincided with the emplacement of the ELIP^9^. Feng et al.^10^ suggested that the thermal pulse event induced by the ELIP accelerated the maturation of source rocks, and resulted in mass high-temperature methane emissions in Southwestern China, which impacted fossil energy formation and episodic carbon changes^10^. We believe that these latest studies further support our interpretation and provide strong evidence that ELIP-related hydrothermal activity caused rapid pyrolysis of paleo-oil pools, strongly questioning the previously established oil cracking timeline used by Huang et al.

## High-temperature methane gas generation and emissions were not overestimated

Approaches suggested by Huang et al., for questioning volume of high-temperature methane generation and emissions are meaningless and invalid, because they are purely based on the concept of industrial natural gas reserves. The extent of methane emissions from large geological domains is far greater than the natural gas reserves used in the oil industry. From the perspective of the organic carbon cycle within the geosphere, the release of methane is of high probability, while the accumulation of methane to achieve industrial grades is of low probability. Petroliferous basins may generate and discharge natural gas at all times, while anomalous tectonic-thermal events often accelerate this process^11^. The magnitude of our estimate of the high-temperature methane emissions seems huge from an industrial perspective, but it is reasonable in the context of the global carbon cycle. Besides, Gou et al.^12^ concluded that, by end of the Permian (primarily occurring during the Ordovician–Devonian), >20% of the oil and >6% of the gas were generated^12^. This supports, rather than contradicts, our estimate that sufficient hydrocarbons could have been generated by the remaining organic matter during the ELIP event, which was significantly delayed and underestimated in previous studies.

The model we used for calculating the volume of high temperature methane followed Xiong et al.^13^. It resulted in the total amount of methane that could ever have been produced from ever overlooked secondary cracking of organic matters under low-temperature metamorphism (<300 °C). In their Matters Arising article, Huang et al. only applies approaches used in oil and gas exploration to judge geological methane emissions which has led to massive underestimation, due to survivorship bias. Oil and solid bitumen used in our paper can include aggregated, scattered, undiscovered, and vanished oil and bitumen. Reservoirs in our paper are geological rather than industrial. They could include liquified hydrocarbons both accumulated and dispersed in all strata (e.g., source rocks, sandstones, and carbonates).

The Sichuan Basin and its adjacent areas are formed by geological processes, such as intense convergence, extrusion, shortening and denudation in later periods. Before the Permian, there existed vast areas of sedimentation and distribution, as well as areas affected by the Emeishan mantle plume. Total area is much larger than the value we used for making our estimation. Our estimates are conservative in terms of stratum thickness, because the reservoirs we are referring to in our paper is geological rather than industrial, which has been misunderstood by Huang et al.

## Uncertainty analysis in the palaeotemperature modeling

Huang et al. mentioned that the maximal burial temperature of the Sinian Dengying Formation was ~250 °C, which was inaccurate. Because, in their cited references, the burial temperature of the Sinian Dengying Formation was concluded as ~220 °C, which is in perfect agreement with our models (Confidence = 95%: 218–222 °C in model MX23 and 210–229 °C in model GS1W) and the literature we cited in this response^5,14,15^, indicating a maximal burial temperature of around 220 °C. This is 20–30 °C lower than our methane clumped temperatures and significantly lower than the temperature of ~296 °C reported by Wang et al.^6^. More importantly, although 220 °C was selected just as a boundary temperature for conditional constraints in our paper, much lower temperatures and larger temperature deficits were expected in the upper reservoirs (e.g., Cambrian). This is because of shallower burial and a reduced geothermal gradient during subsidence following the ELIP event. Huang et al. suggested that thermochemical sulfate reduction (TSR) might cause the formation of dry gas by stripping away wet gas components. However, this contradicts the observation that only small amounts of H_2_S (undetectable to 1.51%) were present as a TSR by-product in the reservoirs^1^. Even trace amount of the TSR reaction products were present, they might have occurred during the ELIP event, and do not have to be generated at a later stage. Therefore, it does not contradict our interpretation.
